# Aggressiveness of care at end of life in patients with high‐grade glioma

**DOI:** 10.1002/cam4.4344

**Published:** 2021-11-09

**Authors:** Rebecca A. Harrison, Alexander Ou, Syed M. A. A. Naqvi, Syed M. Naqvi, Shiao‐Pei S. Weathers, Barbara J. O'Brien, John F. de Groot, Eduardo Bruera

**Affiliations:** ^1^ Department of Neuro‐Oncology University of Texas MD Anderson Cancer Center Houston Texas USA; ^2^ Hospital Corporation of America Healthcare Houston Texas USA; ^3^ Department of Palliative, Integrative, and Rehabilitative Medicine University of Texas MD Anderson Cancer Center Houston Texas USA; ^4^ Department of Internal Medicine University of Kentucky College of Medicine Bowling Green, Kentucky USA

**Keywords:** cancer, end of life, glioma, palliative care, supportive care

## Abstract

**Background:**

Patients with high‐grade glioma (HGG) face unique challenges toward the end of life (EoL), given their aggressive trajectory and neurologic deterioration. Aggressiveness of medical care at EoL has been identified as an important quality metric for oncology patients. At this time, limited data exist around the nature of EoL care of patients with HGG.

**Methods:**

Patients with HGG and palliative care (PC) referral seen between 2010 and 2015 were identified (*N* = 80). Of these, *N* = 52 met inclusion criteria. Random selections of patients with (1) HGG not referred to PC (*n* = 80), and (2) non‐CNS cancers with PC referral (*n* = 80) were identified for comparison. A composite score of aggressiveness of medical care at EoL was calculated for each patient from predetermined variables. A time of eligibility for PC was defined for each patient when predetermined criteria based on symptom burden, functional status, and prognosis were met.

**Results:**

Among the patients analyzed with HGG referred to PC, 59.6% (*N* = 31) were referred as inpatients, and 53.8% (*N* = 28) were referred within the last 12 weeks of life. Patients with HGG had similar aggressiveness of care at EoL regardless of PC referral, and HGG patients had less aggressive care at EoL than patients with non‐CNS cancers (*p* = 0.007). Care was more aggressive at EoL in HGG patients who received late versus early PC referrals (*p* = 0.012). Motor weakness at time of eligibility (OR = 2.55, *p* = 0.002) and more disease progressions (OR = 1.25, *p* = 0.043) were associated with less aggressive care at EoL.

**Conclusions:**

Early clinical‐ and disease‐related features predict the aggressiveness of medical care at EoL in patients with HGG. Formal PC consultation is used infrequently and suboptimally in patients with HGG. Our data suggest that the role of PC in improving EoL outcomes in HGG warrants further evaluation.

## Graphical abstract

Aggressiveness of medical care at end of life (EoL) has been identified as an important quality metric for EoL care; however, to date, this has not been studied in patients with high‐grade glioma. This retrospective study finds less aggressive medical care at EoL in patients with glioma compared with other cancers. We also found that palliative care is often used late and in the inpatient setting in this population, limiting its clinical impact.

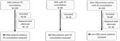

## 1 BACKGROUND

While accounting for <2% of newly diagnosed cancers,^1^ primary brain tumors pose a significant therapeutic challenge in oncology. High‐grade gliomas (HGG), including glioblastoma, anaplastic astrocytoma, and anaplastic oligodendroglioma, are the most common malignant primary brain tumors and impart a unique and devastating symptom burden. These incurable cancers have a median overall survival ranging from 15 months to 5 years for astrocytic tumors,^2^, ^3^ and 15 years for the rarer oligodendroglial tumors.^4^ Their clinical course is often marred by neurologic decline and progressive disability: over half report at least 10 concurrent symptoms, and 40% report their symptoms as moderate or severe.^5^

While research on the quality of end of life (EoL) care in HGG is limited, early evidence suggests that it is markedly inadequate. In a survey of family members of patients with HGG, only 75% felt their affected relative died with dignity, a sequela associated with poor EoL care.^6^ A review of glioblastoma patients at the Memorial Sloan Kettering Cancer Center showed that nearly half were admitted to the hospital within 1 month of death,^7^ with most admissions focused on the management of neurologic decline. A high incidence of late hospitalizations also occur in other nervous system diseases, with a high incidence of in‐hospital death among those with the neurodegenerative disease,^8^, ^9^ and hospice care rates of <1% in patients dying of Parkinson's disease.^8^ This suggests patients with neurologic disease face distinct barriers in attaining effective palliative care (PC) at EoL. The precise nature of these barriers, however, remains undefined.

The period of EoL has received a dedicated study in oncology, and metrics for evaluating the quality of EoL care for cancer patients have been identified.^10^, ^11^ An increase in aggressive and expensive EoL care has been highlighted as a major public health issue in the United States.^12^ Aggressive medical interventions in the last weeks of life, including emergency room visits, hospital and intensive care unit admissions, and chemotherapy administration, are largely considered overly aggressive interventions in this period and indicators of poorer quality EoL care.^10^, ^13^ The National Quality Forum endorses these as quality metrics for EoL care in patients with cancer.^14^ To date, there is limited data on this period of the illness trajectory in patients HGG. Patients with HGG warrant independent study, as their neurologic deficits impart unique EoL care challenges from other cancer patients. Furthermore, the impact of PC involvement on these EoL care outcomes has yet to be explored in this population.

In this retrospective cohort study, we evaluated the aggressiveness of EoL care of patients with HGG and that of other advanced cancer patients with non‐CNS tumors to better understand the quality of EoL care in this population. Within the HGG population, we also studied the association of formal PC consultation with these EoL outcomes and overall survival.

## 2 METHODS

### 2.1 Subjects

The Supportive Care departmental database was used to identify all HGG and non‐CNS cancer patients who received PC consultation, and the MDACC tumor registry to identify those HGG patients without PC referral, between September 2010 and September 2015. Included patients were limited to those diagnosed with cancer in adulthood (>18 years) with a diagnosis of HGG (including anaplastic astrocytoma, anaplastic oligodendroglioma, anaplastic oligoastrocytoma, glioblastoma). Only patients within the seven‐county area of Houston, TX were included to prioritize those patients that would follow longitudinally at our center. Patients excluded from the analysis in the HGG with no PC referral and non‐CNS advanced cancer groups were replaced with randomly selected alternate patients from the database to maintain equitable patient numbers between groups. Once the number of HGG with PC consultation during this defined time period were identified, a random selection of patients with (1) HGG with no PC referral and (2) non‐CNS advanced cancer patients were identified for analysis as well. Patients alive at the time of data analysis were excluded.

Demographic and clinical information was extracted from the medical record. The date of death was extracted from the medical record, Tumor Registry Office, or Social Security Index. Patient symptoms, disease status, and treatment history were analyzed both at the time of diagnosis and at the time of eligibility for PC. Cognitive impairment was defined based on the documentation of its presence in the medical record.

#### 2.1.1 Time of eligibility for PC

The time of eligibility for PC was designated as a time point at which the patient's symptom burden, prognosis, or function indicated that they would strongly benefit from PC intervention. This time point was selected independent of whether a patient had been referred to PC at that time point. It was adapted from previously defined criteria for non‐CNS cancer patients,^12^, ^13^ these include (1) patients with HGG were diagnosed with recurrent/progressive glioblastoma or second recurrence of anaplastic glioma, (2) when life expectancy was <6 months as documented in the chart or in the opinion of the investigators, (3) when the performance status declined below a KPS of 70 or ECOG of 2, or (4) after a hospitalization >5 days for the functional or neurologic decline. This served as a uniform time point derived from patient function, symptoms, and prognosis from which we could analyze the relationship of variables to EoL care delivery.

As an additional variable, the date on which the presence of pain, distress, depressive symptoms, or abuse/neglect was first documented in the medical record.

#### 2.1.2 Aggressiveness of EoL care

The aggressive of EoL care score used has previously been published,^14^ where one point was given for each of the following six indicators in the last 30 days of life: ≥2 ER visits, ≥2 hospital admissions, ≥14 days of hospitalization, and intensive care unit (ICU) admission, death in a hospital, and receipt of chemotherapy within the last 14 days of life. The total score ranges from 0 to 6, with a higher score indicating more aggressive care. In binary analysis, “good” EoL care was defined as a score of 0, where patients had none of the predefined medical interventions defined as overly aggressive.

### 2.2 Statistical analysis

Demographic and clinical data were summarized using descriptive statistics, including means, medians, and range for continuous variables, and frequencies for categorical data. Patient characteristics, symptom burdens, and aggressiveness of EoL care indicators were compared among HGG with PC referrals, HGG without PC referral, and nonneurologic malignancies with PC referrals using Kruskal–Wallis test for continuous variables, and the Chi‐square test or Fisher's exact test for categorical variables. Proportions of patients with early PC referrals were estimated with a 95% confidence interval. EoL outcomes including emergency room visits, hospital and ICU admissions, and late chemotherapy administration were compared between patients with HGG referred to PC and those with other cancers referred to PC, and among patients with early PC referrals (>12 weeks from death) versus later PC referrals (≤12 weeks from death) versus no PC referrals. This definition of early and late PC has been applied in the literature.^15^, ^16^ The logistic regression model was employed to evaluate the association between important covariates and EoL. Kaplan‐Meier method was used to estimate overall survival time and the log‐rank test was used to compare the survival of patients with HGG that received PC referral versus those that did not receive a referral, with the starting time point being the point of eligibility or point of referral for PC. A *p* < 0.05 was considered significant.

The data that support the findings of this study are available from the corresponding author upon reasonable request.

## 3 RESULTS

**FIGURE 1:**
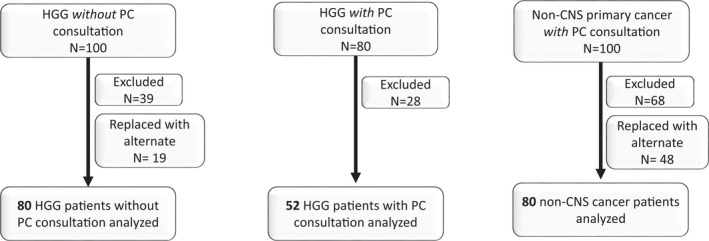
Consort diagram

In total, 80 patients with HGG received PC consultation during this time period, and 52 of these met eligibility criteria. Eighty patients with HGG and no PC consultation and 80 patients with non‐CNS cancer and PC consultation were randomly selected for analysis (Figure 1). Baseline patient characteristics at the time of diagnosis are summarized in Table 1. The mean age at cancer diagnosis of all participants was 57.4 years, and no significant difference in age was noted between groups (*p* = 0.231). Most (87.1%) of the HGG cohort had a diagnosis of glioblastoma, and 3.8% (2/52 patients with HGG referred for PC, all with IDH status known) had an identified IDH mutation.

**TABLE 1:** Patient Characteristics: Demographic and disease‐related characteristics of patients analyzed

Characteristic	All patients	HGG with PC *N* = 52	HGG without PC *N* = 80	Non‐CNS cancer patients *N* = 80	*p*‐value*
Age at cancer diagnosis (years)	57.4	54.9	58.3	58.2	0.231
Gender					
Male	117 (55.2%)	32 (61.5%)	41 (51.3%)	44 (55%)	0.245*
Female	95 (44.8%)	20 (38.5%)	39 (48.8%)	36 (45%)	
Race					
American Indian	17 (8%)	1 (1.9%)	6 (7.5%)	10 (12.5%)	
Asian	1 (0.5%)	1 (1.9%)	0	0	
Black	17 (8%)	3 (5.8%)	4 (5%)	10 (12.5%)	
Hispanic	20 (9.4%)	5 (9.6%)	6 (7.5%)	9 (11.3%)	
White	149 (70.3%)	42 (80.8%)	58 (72.5%)	49 (61.3%)	
Other	8 (3.8%)	0	6 (7.5%)	2 (2.5%)	
Presenting diagnosis					
HGG group					
Glioblastoma	115 (87.1%)	44 (84.6%)	71 (88.8%)		
AO	1 (0.8%)	0	1 (1.3%)		0.648*
AA	16 (12.1%)	8 (15.4%)	8 (10%)		
Clinical trial enrollment					
Yes	73 (34.6%)	14 (26.9%)	32 (40%)	27 (34.2%)	
No	138 (65.4%)	38 (73.1%)	48 (60%)	52 (65.8%)	0.123*
Number of disease progressions	1.9 ± 1.3	1.7 ± 1	1.6 ± 1.3	2.4 ± 1.6	0.003
Performance status (KPS)					
Unknown *n* = 139					
<70	36 (49.3%)	3 (75%)	13 (50%)	20 (46.5%)	
>70	37 (50.7%)	1 (25%)	13 (50%)	23 (53.5%)	0.779

Abbreviations: AA, anaplastic astrocytoma; AO, anaplastic oligodendroglioma; HGG, high‐grade glioma; PC, palliative care.

*Comparing HGG with PC and HGG without PC populations.

### 3.1 Aggressiveness of EoL care

Patients with non‐CNS cancers were significantly more likely to have more than 14 days of hospitalization (*p* = 0.010) or an ICU admission (*p* = 0.049) within the last 30 days of life and were more likely to die in hospital (*p* < 0.001) than HGG patients. Mean EoL care scores were highest for the non‐CNS cancer group (1.34 ± 1.57), reflecting more aggressive care at EoL, compared with the HGG patients that received (0.65 ± 1.1) and did not receive (0.69 ± 1.01) formal PC consultation (*p* = 0.007). Among patients referred for PC, documented clinical trial enrollment (*p* = 0.029) as well as outpatient PC referral (*p* = 0.010) were associated with a lower likelihood of aggressive medical care at EoL (see Table 2).

**TABLE 2:** Variables associated with aggressiveness of medical care at EoL in patients referred to PC

Variable	*N* (%)	Quality of EoL care		*p*‐value
		Good	Poor	
Patient group				0.012*
HGG with PC	52 (39.4)	35 (67.3%)	17 (32.7%)	
Non‐CNS cancer with PC	80 (60.6)	36 (45%)	44 (55%)	
Gender				
Male	76 (57.6%)	40 (52.6%)	36 (47.4%)	
Female	56 (42.4%)	31 (55.4%)	25 (44.6%)	0.756
Clinical trial enrollment				
Yes	41 (31.3%)	28 (68.3%)	13 (31.7%)	
No	90 (68.7%)	43 (47.8%)	47 (52.2%)	0.029*
Prior bevacizumab therapy				
Yes	35 (67.3%)	26 (74.3%)	9 (25.7%)	
No	17 (32.7%)	9 (52.9%)	8 (47.1%)	0.124
Referral site to PC				
Inpatient	64 (48.5%)	27 (42.2%)	37 (57.8%)	
Outpatient	68 (51.5%)	44 (64.7%)	24 (35.3%)	0.010

Note

“Good” denotes those patients with an EoL care score of 0, not meeting any of the predetermined criteria for overly aggressive care at EoL; “poor” denotes patients having ≥1 of the predetermined criteria.

Abbreviation: EoL, end of life.

Those variables associated with aggressiveness of EoL care are presented in Table 3 for all groups. On the evaluation of all patients using multicovariate logistic regression, patients with weakness at time of eligibility were over twice as likely to have less aggressive EoL care (OR = 2.55, *p* = 0.002), and a higher number of disease progressions was associated with a 25% increase in the chance of good EoL care (OR = 1.25, *p* = 0.043). In analyzing those patients that saw PC univariately, more severe symptoms related to appetite (*p* = 0.049) and shortness of breath (*p* = 0.006) on the ESAS (Edmonton Symptom Assessment System) were associated with less aggressive EoL care, and higher pain scores (*p* = 0.058) approached significance. Within this analysis, patients with HGG had lower mean scores than the non‐CNS tumor group on scales of shortness of breath (1.77 ± 2.72 [*n* = 35] vs. 3.67 ± 3.43 [*n* = 70]), appetite (3.11 ± 3.21 [*n* = 35] vs. 5.36 ± 3.17 [*n* = 67]), and pain (3.56 ± 3.8 [*n* = 36] vs. 4.91 ± 3.02 [*n* = 70]). Of presenting symptoms, motor weakness (*p* = 0.006) was associated with better quality of EoL care, whereas seizure, headache, sensory deficit, bulbar symptoms, and cognitive complaints were not. Patients with an inpatient PC referral were 70% less likely to have a good quality of EoL care than those receiving ambulatory referrals (OR = 0.307, *p* = 0.013), and motor weakness remained the only clinical symptom at the time of eligibility for PC that was associated with good quality of EoL care (*p* = 0.006) from multicovariate logistic regression.

**TABLE 3:** Predictors of aggressiveness of care at EoL identified on multivariate logistic regression

Variable	Odds ratio	*p*‐value
All patients		
Number of disease progressions	1.249 (1.007–1.549)	0.043*
Weakness at time of eligibility	2.546 (1.407–4.610)	0.002*
All patients referred to PC		
Inpatient versus Outpatient referral site	0.307 (0.122–0.775)	0.013*
ESAS shortness of breath	0.862 (0.754–0.986)	0.031*
Weakness at time of eligibility	4.635 (1.545–13.909)	0.006*

Abbreviations: EoL, end of life; ESAS, Edmonton Symptom Assessment Symptom.

### 3.2 Timing of PC referral

The time between PC referral and death was not significantly different between HGG (10.7 weeks) and non‐CNS cancer patients (13.1 weeks, *p* = 0.746). Within the HGG population, earlier referral to PC (≥12 weeks before death) was associated with a significantly better composite score for the aggressiveness of EoL care than late referral (*p* = 0.012). When evaluating the latency between the first time pain, distress, depressive symptoms, neglect, or abuse were documented in the chart and PC referral, there was a nonsignificant trend toward shorter latency in HGG patients (45.5 ± 76.6 weeks) compared with non‐CNS cancer patients (75.8 ± 129 weeks, *p* = 0.277). Of the HGG patients that received SC, 31 (59.6%) were referred to as inpatients and 21 (40.4%) as outpatients. Among our non‐CNS cancer population, 33 (41.3%) were referred as inpatients and 47 (58.8%) in the ambulatory setting. Over half of HGG patients (*n* = 28, 53.8%) had late PC referral (≤12 weeks until death).

All patients were deemed eligible for PC based on our “time of eligibility” criteria. The mean number of weeks between the time of eligibility and death was 37.94 ± 39.77 weeks for all HGG patients, and there was no significant difference in this latency between patients referred and not referred to PC (*p* = 0.805).

### 3.3 Decision‐making capacity

Of all HGG patients, only three did not have clear documentation of medical decision maker in the chart at the time of eligibility for PC (*n* = 52, 100%, and *n* = 77, 96.3% of those referred and not referred to PC, respectively, had documentation). Of patients with surrogate decision makers used at the time of eligibility for PC, 52.0% (*n* = 40/77) were found to have less aggressive EoL care, whereas 75.0% (*n* = 39/52) of those who were making autonomous decisions at this time had less aggressive EoL care (*p* = 0.008). At the time point, where medical decision‐making was extracted (time of eligibility), 73.5% (*n* = 97/132) HGG patients had clinical documentation of cognitive impairment, with similar proportions within the groups that received (69.2%, *n* = 36/52) and did not receive (76.3%, *n* = 61/80) PC.

### 3.4 Survival

There was no significant difference in overall survival time from time of eligibility for PC in HGG patients who did (median overall survival time 25.2 weeks) and did not (26.9 weeks) receive PC referral (*p* = 0.743).

## 4 DISCUSSION

Our study provides novel insight into the EoL period in patients with HGG, a stage of illness that has not been well‐explored in this population. We identified several novel findings: a small number of HGG patients received formal PC consult, and when made, it is often late in the illness trajectory. We found that a significant portion of this population is receiving acute or aggressive anticancer care toward the EoL, a practice considered the poor quality of EoL care.^14^, ^17^ Notably, there are early clinical‐ and disease‐related features that can predict the quality of EoL care, suggesting certain subpopulations of patients can be identified as being at risk and may benefit from earlier intervention.

The central purpose of this study was to evaluate the impact of PC on the aggressiveness of medical care at EoL in patients with HGG. Our analysis found no difference in the aggressiveness of care at EoL in patients who had a PC referral in comparison with those who did not. This is contrary to prior literature in non‐CNS cancer patients, which shows PC is associated with a lower likelihood of in‐hospital death and less chemotherapy use in advanced disease.^18^, ^19^ This may reflect the manner PC was implemented in our study population. While nearly all patients with HGG were deemed eligible for PC referral by our predetermined criteria, a small minority were in fact referred. These patients may be inherently different: high early symptom burden, social challenges, and difficult communication around goals of care may all have prompted these selected referrals. This context may limit the positive impact of PC on clinical outcomes. Furthermore, a large proportion of patients were referred late in the illness course and in the inpatient setting, both of which are associated with poorer EoL outcomes.^20^ These findings suggest that while PC was incorporated in the care of select patients with HGG, it was not implemented in a way that optimized impact.

Brain tumor patients have a distinct disease trajectory and symptom constellation, prompting our comparative evaluation between patients with HGG and non‐CNS cancers. We found that HGG patients had a better quality of EoL care than non‐CNS cancer patients. This finding may reflect clinical distinctions between HGG and other non‐CNS cancers. There are no curative treatments in HGG, and most subgroups of HGG have an overall survival ranging from 1 to 5 years,^2^ and limited available treatments, while our non‐CNS cancer population had more heterogeneity in regards to their prognosis at the time of cancer diagnosis. This may lead to tempered patient and physician expectations of antitumor therapy toward EoL. The nature and severity of early symptoms in HGG patients may also be contributory, as presenting symptoms often affect cognitive, language, sensory, and motor functions that are central to their independence. Up to one‐third of patients with glioblastoma present with a low‐performance status,^21^ implying that they need assistance in daily life, while patients with non‐CNS cancers are generally more functionally intact at diagnosis.^22^ In addition, HGG patients report an early and negative impact on quality of life.^23^ These distinctions may prompt a preference to focus on the quality of life and interest in limiting medical intervention that would not contribute meaningfully to this.

We identified various clinical‐ and disease‐related features predictive of quality of EoL care in HGG. Symptoms at the time of diagnosis were predictive of quality of EoL, as a presentation with motor weakness was associated with better quality of EoL care. Evaluating symptoms at the time of eligibility for PC, the presence of weakness remained associated with good EoL care. The association of this symptom with better EoL outcomes may be for several reasons. Motor weakness is strongly associated with functional independence^24^ and has been found to be is the most common reason for hospital admission and for symptom management in patients with primary brain tumors.^25^, ^26^ The finding of weakness at the time of eligibility being associated with a 2.5‐fold increase in odds of less aggressive care at EoL. The nature of this relationship warrants further investigation, to better determine the contributions of patient and physician preferences, behaviors, and perceptions to this distinction.

A distinctive symptom in HGG patients is early cognitive impairment. While only 6.9% had cognitive impairment at the time of eligibility for PC in the non‐CNS cancer population, 75.1% of those with HGG were noted to have cognitive impairment at this time point. In the cancer population at large, impaired decision‐making has been found to be underdetected by physicians.^27^ A core element of PC is the discussion and clarification of advanced directives. Advanced care planning increases the likelihood that the person is dying in their preferred place and out of the hospital, at EoL,^28^ and reduces hospital readmission and ICU utilization at EoL.^29^ While we did not find a relationship between cognitive impairment and quality of EoL, we did note that the patients who served as autonomous decision makers were more likely to have a good quality of EoL (75%) than those who had an active surrogate decision maker, for whom only 50% had a good quality of EoL. Much of the study around cognitive impairment and relationships with surrogate decision makers have been in the dementia population, where the focus is on overall goals, medical care, and day‐to‐day life.^30^ This, however, is distinct from the process in patients with HGG, who have a more aggressive clinical trajectory, and are faced with medical decisions around toxic treatments. As such, regular and routine assessment of capacity is of increased importance in HGG patients.

The treatment course of patients with HGG appears to influence care at EoL. A higher number of disease recurrences/progressions was associated with less aggressive medical care at EoL. The driver for this observation is unclear: it may reflect an increased comfort with accepting cessation of anticancer treatment when a greater number of therapies have been attempted, or the opportunity for a greater therapeutic alliance between patient and physician after more recurrences/progressions have been tackled. Notably, participants in clinical trials were found to have less aggressive medical care at EoL than nonparticipants in the HGG population. Clinical trial participation is associated with incorporating novel and experimental therapies, which may be considered a more aggressive approach to anticancer therapy. There are, however, many other factors associated with trial participation, including patient trust in the medical system, medical literacy, socioeconomic status, educational background, and health care literacy.^31^, ^32^, ^33^ These may all influence or be interrelated with the patient's own understanding of their illness, expectations of therapy, and trust in their oncologist. The physician approach to these patients may be different as well: in an effort to establish realistic expectations of treatment, discussions around goals of care may tend to occur earlier in this population.

This hypothesis‐generating study presents novel information regarding how our patients with HGG are cared for at the EoL and the potential impact of PC on these outcomes. There are limitations to this study. The retrospective nature of the study imposes inherent limitations. A small minority of patients with HGG were referred for PC at our institution, limiting the overall patient numbers in this study, and also introducing the possibility of selection bias. We also feel it is important to highlight that death in the hospital, one of the metrics used to characterize overly aggressive EoL care, may be beneficial to select patients with high levels of distress,^34^ highlighting the limitation of applying this quality metric to all patients. Despite these limitations, this study provides novel insight into the EoL period in patients with HGG and identifies various demographic, disease‐related, and clinical variables that can influence EoL care in this population. It also generates further questions about the optimal implementation of supportive care in this population to maximize its clinical impact. Further prospective study of the EoL period and the implementation of PC is needed in patients with HGG to best care for this population as they approach the EoL.

## ETHICAL CONSIDERATIONS

This study was approved by the Institutional Review Board of the University of Texas MD Anderson Cancer Center (protocol PA17‐0453).

## CONFLICT OF INTEREST

The authors of this manuscript have no conflicts of interest to report.

## Data Availability

The data sets analyzed during the current study are available from the corresponding author on reasonable request.
